# On-farm biosecurity practices and causes of preweaning mortality in Canadian commercial mink kits

**DOI:** 10.1186/s13028-017-0326-8

**Published:** 2017-09-08

**Authors:** Nicole Compo, David L. Pearl, Brian Tapscott, Amanda Storer, Jutta Hammermueller, Marina Brash, Patricia V. Turner

**Affiliations:** 1 Department of Pathobiology, Ontario Veterinary College, University of Guelph, Guelph, ON N1G 2W1 Canada; 2 Department of Population Medicine, Ontario Veterinary College, University of Guelph, Guelph, ON N1G 2W1 Canada; 3 Ontario Ministry of Agriculture, Food, and Rural Affairs, 6484 Wellington Road 7, Unit 10, Elora, ON N0B 1S0 Canada; 4 Animal Health Laboratory, University of Guelph, Guelph, ON N1G 2W1 Canada

**Keywords:** Mink production, Mink disease, Salmonellosis, Zoonoses

## Abstract

**Background:**

Mink are an important animal commodity group in Canada and excessive kit mortality represents a significant loss to production. National biosecurity standards have been developed for Canadian mink farms, but it is unclear how well these standards have been implemented as there are no studies correlating management practices of mink producers with causes of death in mink kits. To that end, we surveyed Ontario mink producers on their biosecurity and management practices and conducted almost 5660 post mortem examinations on found-dead, preweaned kits to characterize mink farm biosecurity practices and causes of death in preweaned kits.

**Results:**

We found that very few biosecurity and management practices were uniformly used by producers, despite good awareness of appropriate practices. Use of personal protective equipment was implemented by fewer than 50% of respondents, while control of mink shed access, disinfection of feed containers after use, and use of a rodent control program were the only practices implemented by greater than 70% of respondents. Only 18% of producers reported regular use of antimicrobials in feed or water, although 91% stated they used antimicrobials for treatment of bacterial diseases on a regular basis. On post mortem examination, no gross abnormalities were noted in 71% of the kits, 45% were thought to be stillborn or aborted, 27% had some form of abnormal fluid distribution in the body, and 2% had a congenital malformation. A subset of 69 gastrointestinal tract samples was submitted for bacterial culture, of which 45 samples yielded sufficient growth. Most interesting was the identification of *Salmonella enterica* serovar Heidelberg in 11% of samples.

**Conclusions:**

The results of this study will provide a benchmark for Canadian mink producers and their veterinarians, defining the areas to which greater attention should be given to ensure more rigorous biosecurity practices are in place. Ultimately, these improvements in practices may contribute to increased mink production and animal well-being.

**Electronic supplementary material:**

The online version of this article (doi:10.1186/s13028-017-0326-8) contains supplementary material, which is available to authorized users.

## Background

Mink are purpose bred for their pelts and are an important animal commodity group in Canada. In 2014, there were approximately 784,000 breeder mink on 237 mink farms across Canada, with more than 3.2 million pelts sold and valued at almost $98 million [1]. While most costs to producers are relatively fixed, mink mortality can be highly variable and it can represent a significant loss to productivity. Published data are lacking in this area; however, empirical evidence suggests that early loss of preweaned kits likely represents the largest area of overall mortality on mink farms.

Historical preweaning mortality rates on mink farms range from 20 to 25% in Canada and Argentina, respectively [2, 3], somewhat higher than that noted for other food animal commodity groups, such as swine at 15% [4], but similar to meat rabbits [5, 6]. For the most part, post mortem examinations are not conducted routinely on found dead mink kits and a cause of death is not identified. Even in studies specifically evaluating mortality in mink kits, gross lesions were not present in up to 78% of animals dying within 4 days of birth [2]. Two previous studies identified systemic infection as the most common cause of death in unweaned mink kits >4 and 7 days of age, respectively, although specific diseases were not further characterized [2, 3]. Other gross pathology findings in these studies included evidence of starvation, hypothermia, dystocia, anasarca, and congenital defects [2].

The Canadian Food Inspection Agency has indicated that the most serious infectious diseases that mink producers face in Canada are Aleutian disease, mink enteritis virus, distemper, and hemorrhagic pneumonia due to *Pseudomonas aeruginosa* [7]. Potential sources of infectious agents vary by disease. For example, antibodies to Aleutian disease have been identified in skunk, wild mink, badgers, and small rodents [8–10], while foxes were the source of a major distemper outbreak on Danish mink farms between July 2012 and February 2013 [11, 12]. Poorly managed manure and carcasses can contribute to the persistence of pathogens, such as mink enteritis virus, a parvovirus, which remains infectious in the environment for at least 9 months [13]. Newly purchased mink stock, even when apparently healthy, can also harbor and shed mink enteritis virus in the feces for at least 1 year [13]. Feed and water sources have also been implicated, for example, an outbreak of swine influenza in Danish mink was determined to arise from feed containing infected swine lung tissue [14]. Additional sources of infectious agents include humans, companion animals, vehicles, and farm equipment [7].

Biosecurity resources are available to producers to help limit spread of disease between farms and wildlife, and to minimize on-farm losses. There are two guides produced for Canadian mink farmers, the *Code of Practice for the Care and Handling of Farmed Mink* [15], and the CFIA National Farm-Level Mink Biosecurity Standard [7]. The CFIA defines on-farm biosecurity as, “a set of well-organized, well-planned procedures that are applied at the farm level,” with the intent to, “reduce exposure of mink to infectious disease-causing agents, including their introduction, spread within the farmed mink population and release from the farm,” [7]. This document introduces standards based on well-known principles of isolation, sanitation, traffic control, herd health management, and maintenance of the biosecurity program once it is established. The standard provides basic and voluntary recommendations, which establish target outcomes on access management, animal health management, and operational management [7]. Although the document is readily available, it is unclear to what extent the standards are implemented at the farm level in Canada.

To better characterize causes of production losses on Canadian mink farms as well as implementation of new biosecurity guidelines, the goals of this study were twofold: (1) to determine the causes of death in found dead preweaned mink kits, and (2) to characterize uptake of on-farm biosecurity and management practices on mink farms.

## Methods

### Part I—preweaned kit mortality surveillance

#### Farm recruitment and sample collection

Between April 1 and 30, 2013, all mink producers in Ontario were contacted through the Ontario Fur Breeders Association and the Ontario Ministry of Agriculture, Food and Rural Affairs (OMAFRA) for enrollment and a total of 21 of 44 producers were enrolled. Participating producers were provided with supplies, including instructions for cadaver collection and storage, freezer bags, plastic totes, and permanent markers. Producers were instructed to collect dead kits daily into dated, labeled freezer bags, and to store frozen at −20 °C until pick up. Frozen, dead kits were collected at three and 6 weeks post-whelping from participating farms. Depending on the number of dead kits collected, a proportion of or all dead kits were collected. Farm and kit information were linked using anonymized identification codes for each farm. Dead mink kits between 0 and 45 days of age were collected and returned to the University of Guelph and stored at −20 °C until processed.

#### Sample processing

Bags of cadavers from each farm were thawed at 4 °C, cadavers were weighed, and gross post mortem examinations were conducted in the Dept. of Pathobiology, University of Guelph by veterinary pathologists or trained technical personnel (PVT, MB, AS) on a total of 5657 kits, and abnormalities were recorded. Samples of sections of gastrointestinal tract from 69 kits with suspected enteritis, based on gross examinations, and sections of skin from three kits with dermatitis, were submitted for aerobic microbiologic culture to the Animal Health Laboratory, University of Guelph, following standard procedures.

#### Preweaning mortality survey

Participating mink farms were contacted by OMAFRA by email or telephone and asked to submit production figures for the 2013 whelping season. Producers were asked to include the total number of kits born on the farm, how or when this count was performed (i.e., actual counts versus estimates and on what day the count occurred post-whelping), how many kits were weaned, and at what age kits were weaned. This information was used to calculate the self-reported incidence of preweaning mortality from all causes per participating farm.

#### Environmental data

To determine whether there were any significant weather variations during the whelping season, daily environmental parameters were collected during the whelping season from Environment Canada (https://weather.gc.ca/canada_e.html) for the four counties encompassing the study farms: minimum and maximum daily temperatures, total precipitation, and highest relative humidity.

#### Statistical analyses

Statistical analyses were conducted using Stata (StataSE 14, College Station, TX). For post mortem results, models concerning the following outcomes were fitted using multilevel logistic regression: breath taken (yes/no), evidence of food in gastrointestinal tract (yes/no), presence of any congenital lesion (yes/no), presence of any abnormality (yes/no; including any congenital lesions, abnormal fluid distribution, signs of trauma, and/or evidence of dystocia), and producer-reported incidence of preweaning mortality. These models were used to examine statistical associations between the above dependent variable and the following independent variables: total kits born, average age weaned (in days), and when kit counts were taken (reported in days post-whelping). Random intercepts were included for farm and shed, except for mortality, for which only data on farm of origin were available. For postmortem body weight (in grams), the same associations were examined, except multi-level linear regression models were fitted. The assumption of linearity between independent and dependent variables (in the log odds scale for logistic regression models) was examined using lowess curves (i.e., locally weighted regression). If the assumption was violated, a quadratic term was included, if appropriate, or the independent variable was modeled as a dichotomous variable (i.e. above and below a median cut-off). For all outcomes, intercept-only multi-level logistic and linear models were fitted to estimate variance components and to calculate the variance partition coefficient at the farm, shed and kit level. To estimate the variance at the kit level for our multi-level logistic regression models, the latent variable technique was used [16]. Significance level for all analyses was set at α = 0.05.

### Part II—survey of husbandry and management practices

In April, 2014, a detailed survey regarding farm practices was sent by email or post, depending on the producer’s preference, to 26 Ontario mink producers of which 11 responded. Participants were informed prior to enrollment of the survey’s purpose and that any identifying information would be confidential. Five of the survey respondents had participated in the preweaning mortality project and six respondents had not. The survey included questions on owner demographics (e.g., years in operation, number of employees, etc.); proximity of farm/mink to other livestock; water and feed source treatment; animal health, quarantine and carcass disposal; biosecurity and hygiene practices; and, manure disposal and pest control (see Additional file 1). Some questions had open-ended answers, while others required selection from a fixed list of choices. In all cases, participants had an option to supply their own answers if they did not feel the options provided adequately described their farm’s practices. Additionally, participants were informed that they could refuse to answer any question and still remain in the study. Once completed, participants were instructed to return the survey via email or fax. Finally, participants were informed that they could withdraw their survey information at any time by contacting the study coordinator. The project was reviewed and approved by the University of Guelph Research Ethics Board (14MR013).

#### Statistical analyses

Statistical analyses were conducted using Stata. For descriptive statistics concerning answers to survey questions which could be answered as yes/no proportions and their exact 95% confidence intervals are reported. For descriptive statistics concerning answers to survey questions which had continuous answers, median, range, mean, standard deviation and their exact 95% confidence intervals are reported. Using exact univariable logistic regression, associations between the use of each biosecurity and management practice measure were examined and the following independent variables evaluated (dichotomized as above/below the median): number of years the producer had been in operation, the number of adult females on farm, and the number of years females were kept for breeding. The significance level for all analyses was set at α = 0.05.

## Results

### Part I: preweaned kit mortality surveillance

**Fig. 1:**
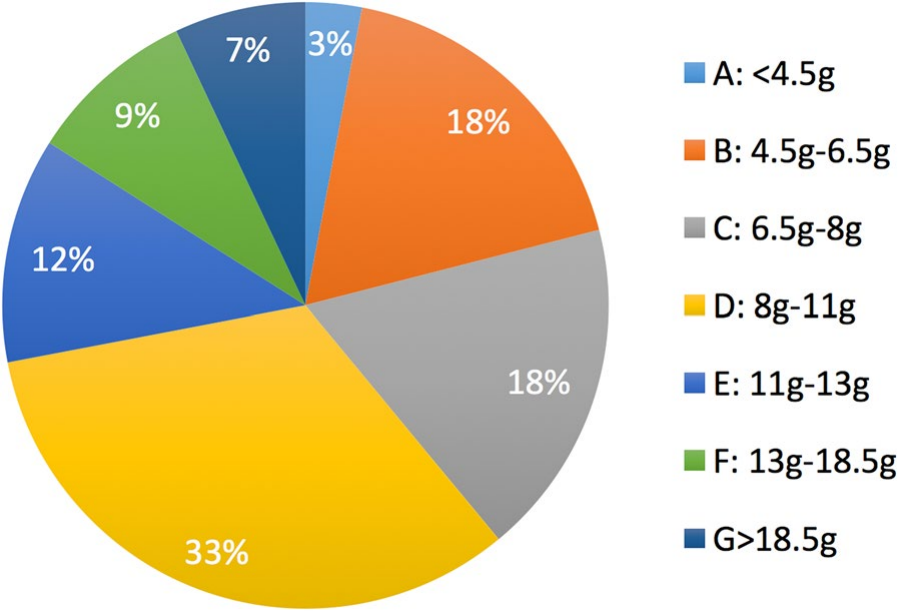
Weight distribution of preweaned, found dead mink kits at the time of post mortem examination from 21 fur farms in Ontario (n = 5659)

A total of 5657 post mortem examinations were completed on found dead preweaned mink kits, and of these, 11 kits were excluded from the final analysis due to incomplete data. Distribution of kits based on weight category are summarized in Fig. 1; 55.4% were estimated to have breathed prior to being found dead, based on lung flotation. Overall, 66% of kits born dead were <11 g in weight, 32% were between 11 and 18.5 g and just 2% were >18.5 g. As weight increased, kits were more likely to have taken a breath (OR = 1.03, P < 0.001. Of kits born alive, 11% had food in their gastrointestinal tract at the time of death, representing 6.0% of all animals examined. No gross abnormalities were present in 70.6% of animals (Table 1).

**Table 1:** Prevalence of gross post mortem lesions in 5657 preweaned, found dead mink kits from 21 commercial mink farms in Ontario

Diagnosis	Overall prevalence (%)	95% CI
Breath taken	55.4	54.1 to 56.7
Evidence of dystocia	1.0	0.7 to 1.3
Trauma	1.3	1.0 to 1.6
Food in gut	6.0	5.4 to 6.6
Abnormal fluid distribution	26.5	25.4 to 27.7
Hydrothorax	21.5	20.4 to 22.5
Hydroperitoneum	16.1	15.2 to 17.1
Subcutaneous fluid	11.7	10.9 to 12.6
Anasarca	1.2	0.9 to 1.5
Congenital lesions	2.0	1.7 to 2.4
Cleft palate	0.9	0.7 to 1.2
Umbilical hernia	0.2	0.1 to 0.4
Other	0.9	0.6 to 1.1
Any abnormality	29.4	28.2 to 30.6

The percent variance for the outcomes ‘breathed,’ ‘food in gastrointestinal tract,’ ‘congenital lesion,’ ‘any abnormality,’ and ‘weight’ explained at the kit level ranged from 78 to 86%. Much less variance was explained at the farm level (11 to 20%) and almost no variance explained at the shed level (0 to 5%). This indicates that most of the variance in these outcomes is explained at the kit level rather than the farm or shed levels.

Approximately 1% of kits examined had abnormalities consistent with dystocia during birth. Signs of trauma were noted in 1.3% of animals, although in some cases it was not possible to discern if the injuries were sustained ante or post mortem. Four abnormal fluid distribution patterns were commonly observed, including hydrothorax, hydroperitoneum, subcutaneous edema, and anasarca, a severe form of generalized edema. When considered together, 26.5% of kits had some form of abnormal fluid distribution (95% CI 25.4 to 27.7%) (Table 1).

Congenital malformations, specifically, cleft palate, umbilical hernia, or other (i.e., low incidence conditions such as heart defects) and were seen in 0.92, 0.87, and 0.23% of animals, respectively (Table 1). When considered together, congenital abnormalities were present in 2% of all found dead preweaned kits (95% CI 1.7 to 2.4%).

In suspected enteritis cases (n = 69), gastrointestinal tract samples were taken and submitted for bacterial culture. The proportion of samples yielding significant (3+ to 4+) growth of 1, 2, 3 or 4 bacterial species was 23, 20, 16, and 1%, respectively and 24 samples had minimal or no growth and were excluded from the final analysis. Ten bacterial species were isolated from the 45 samples that yielded sufficient growth (Table 2): five species from *Enterococcus*, two species from *Staphylococcus,* and one species each from *Escherichia, Salmonella* and *Streptococcus*, which represent a variety of gastrointestinal tract commensal and potentially pathogenic or zoonotic organisms. *Salmonella* spp. was isolated from four different farms, and one farm yielded two separate positive samples. One skin sample from the three suspected dermatitis cases yielded 4+ growth of *Staphylococcus delphini*.

**Table 2:** Bacterial culture results from 45 found dead kits with suspected enteritis

Bacteria cultured	No. of samples (%; 95% CI)^a^
*Enterococcus faecalis*	30 (67; 51 to 80)
*Escherichia coli*	16 (36; 22 to 37)
*Enterococcus faecium*	10 (22; 11 to 37)
*Staphylococcus delphini*	10 (22; 11 to 37)
*Enterococcus hirae*	5 (11; 4 to 24)
*Salmonella enterica* serovar Heidelberg	5 (11; 4 to 24)
*Enterococcus gallinarum*	3 (7; 1 to 18)
*Enterococcus avium*	3 (7; 1 to 18)
*Streptococcus canis*	1 (2; 0.1 to 12)
*Staphylococcus sciuri*	1 (2; 0.1 to 12)

^a^Percentages do not equal 100 as some samples had multiples species present. Only 45 samples yielded sufficient growth for inclusion

Fifteen of 21 farms participating in the mortality surveillance study provided data on the total number of kits born, the day this number was counted (i.e., # of days post-whelping), total number of kits weaned, and the average day of weaning (Table 3). There was a statistically significant quadratic relationship between kit body weight at the time of post mortem examination and the day on which kits were counted, (main effect: coefficient = −2.75, 95% CI −9.0 to 3.5, P = 0.391; quadratic effect: coefficient = 0.33, 95% CI 0.01 to 0.65, P = 0.042). Initially, body weight decreased as the day counted increased, but after 5 days, body weight increased with increasing day counted. Additionally, and not surprisingly, the odds of a kit having food in its gastrointestinal tract at post mortem examination was significantly increased if the day kits were counted exceeded 3 days post-whelping (OR = 3.86, 95% CI 1.05 to 14.0, P = 0.042). The odds of detecting a kit having any congenital lesion was significantly increased with each day increase following birth (OR = 1.07, 95% CI 1.01 to 1.12, P = 0.013). No other associations tested were statistically significant.

**Table 3:** Producer-reported production characteristics and incidence of mortality from 2013 (results from 15 of 21 participating farms)

Parameter	Median	Range	Mean	SD	95% CI
Total kits born	13,698	2900 to 27,855	13,860	6713.1	9983.6 to 17,735.7
Day counted (post-whelping)	3	0 to 21	5.3	5.7	2.0 to 8.6
Total kits weaned	11,250	2700 to 26,000	11,995	6181.7	8425.6 to 15,564
Average age weaned (days)	56	42 to 75	59.3	10.2	53.6 to 64.9
Incidence of mortality (%)	6.8	3.1 to 15.4	7.8	3.8	5.5 to 10.1

No conclusions could be drawn relating kit mortality to general weather patterns and there were no environmental extremes in overall temperature, precipitation, and relative humidity patterns documented during the preweaning period for any of the counties monitored (data not shown).

### Part II: survey of husbandry and management practices

Of 26 biosecurity surveys distributed to Ontario mink farms, 11 were completed and returned (42% completion rate, or a 25% response rate for all mink farms in Ontario).

#### General farm information

Farm demographics are summarized in Table 4. Farms had generally been in operation for many years, indicating that the mink industry is not a growth industry, as no new farms are being established. In terms of the number of breeding females kept on farm, farms were not generally very large. Employees working on the farm, including family members, were categorized as full time (FT), part time summer (PTS) and part time pelting (PTP), with farms having more PTP employees than FT or PTS (Table 4). There was a wide range in number of pelts produced (range 2800 to 36,000) and total kits born (range 3200 to 39,000) across Ontario mink farms in 2013 (Table 4). The average number of years that breeding females were kept was 2.7 (range 2 to 4, median = 3), while for breeding males, it was 1.5 years (range 0 to 3, median = 1).

**Table 4:** Producer-reported farm demographics from biosecurity survey (n = 11)

Parameter	Range	Mean	Median	SD	95% CI
Farm size (# breeding females)	700 to 6900	3066	3400	2022	1708 to 4425
# of years in operation	15 to 85	59.5	67	24.7	43.0 to 76.1
# of full-time employees	0 to 11	5.2	6	3.7	2.7 to 7.7
# of part-time summer employees	0 to 11	2.5	1	3.4	0.2 to 4.73
# of part-time employees at pelting	0 to 20	5.6	3	5.6	1.3 to 8.9
# of pelts produced (2013)	2800 to 36,000	14,668	16,900	10,734	7457 to 21,879
# of kits born (2013)	3200 to 39,000	5948	17,500	11,648	8123 to 23,774

All producers reported being within 3 km of other agricultural species, including other mink farms, chickens, dairy and beef cattle, swine, and turkeys. Only four producers (4 of 11; 36%; 95% CI 11 to 69%) owned other production species, including broiler chickens, beef cattle, and horses. Most producers (9 of 11; 81%; 95% CI 48 to 98%) visited other farms, although not often (less than monthly), and most producers also reported having visitors from other farms, which included mink, beef and dairy cattle, and swine operations.

All but one farm reported receiving farm water from wells (including deep, drilled, and shallow, dug wells) (95% CI 58.7 to 99.7%), with one farm using exclusively municipal water and one farm using both wells and municipal water. Six of 11 (55%; 95% CI 23.4 to 83.3%) farms tested the water for bacteria, with 50% testing on an annual basis, and 50% testing less than annually. Four of 11 farms (36%; 95% CI 10.9 to 69.2%) treated the water on-farm by a variety of methods, including dechlorination, use of an injection pump, hydrogen peroxide or descaling.

Six of 11 (55%; 95% CI 23.4 to 83.3) farms had a perimeter fence surrounding mink sheds and 4 of 6 reported that the fence was at least 6 feet high. Eight of 11 (72%; 95% CI 39 to 94) farms used wet feed made on site, while the remaining 3 of 11 (27%) purchased wet feed, and no producers reported feeding pellets. Only 1 of 11 (9%) producers reported having cement floors in mink sheds, while the remaining 10 of 11 (91%) had packed soil.

#### Animal health responses

**Fig. 2:**
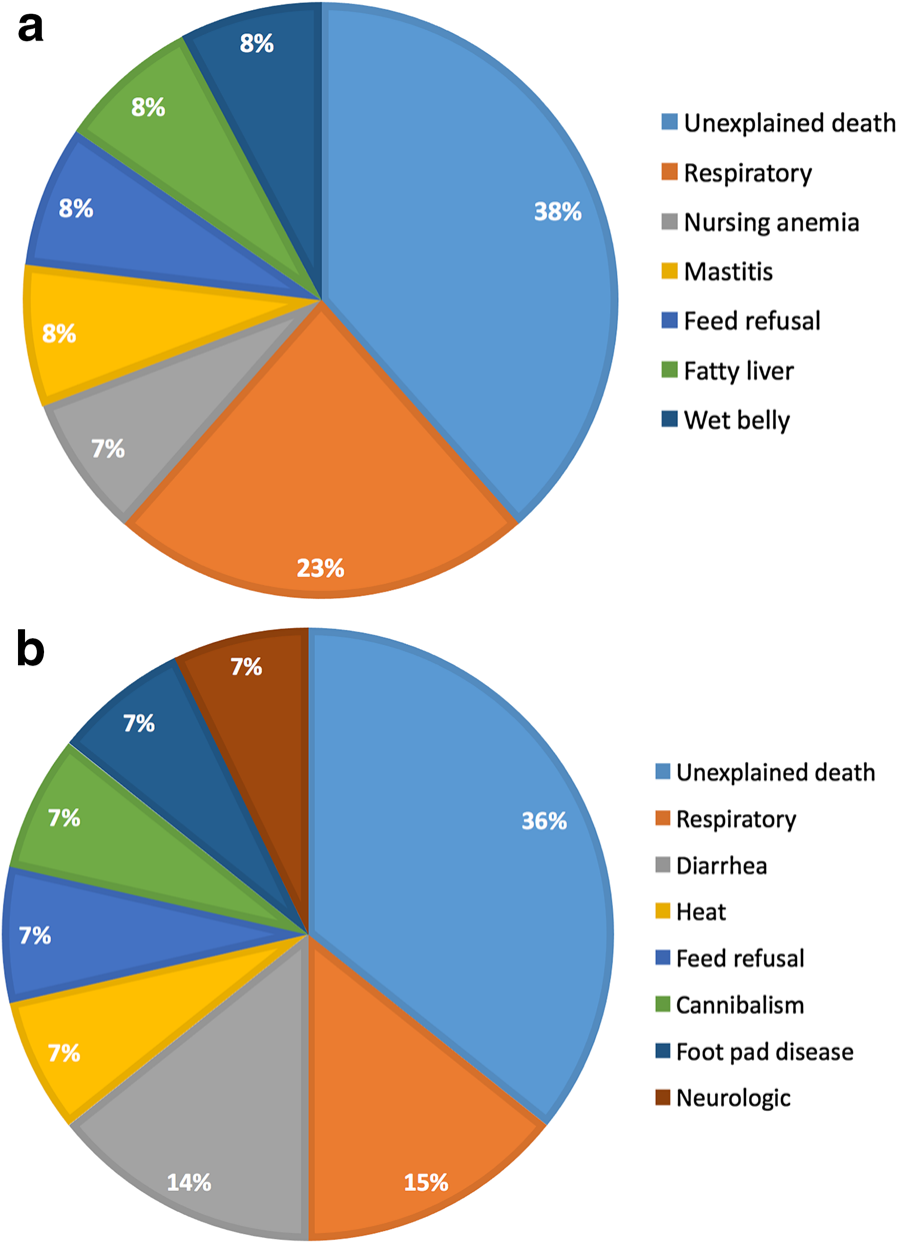
Producer-reported common health problems as answered on biosecurity survey (n = 11). Percentages represent the number of producers identifying the condition as a problem of the total number of responses provided by producers

A variety of health problems for both kits and adult females were reported to be seen commonly by producers (Fig. 2). Ninety-one percent of producers (10 of 11; 95% CI 58.7 to 99.7) responded that they used antimicrobials to treat disease of suspected bacterial origin. Various formulations were reported, which most frequently fell into the penicillin class, used either alone or as a combination product (9 of 11; 82%; 95% CI 48.2 to 97.7%). Other classes of antimicrobials used alone or as a combination product, included macrolides, sulfonamides tetracyclines, and fluoroquinolones (4 of 11; 36%; 95% CI 10.9 to 69.2%) and polypeptides (3 of 11; 27%; 95% CI 6.0 to 61.0). Two producers reported using an antimicrobial combination product that included a penicillin, tetracycline, and sulfonamide. Only 2 of 11 producers (18%; 95% CI 2.3 to 52.8%) reported using antimicrobials in the feed or water regularly.

One producer reported increased mortality in preweaned kits, one producer reported increased mortality in weaned kits, and one producer reported increased mortality in both preweaned and weaned kits in 2013 compared to previous years. Estimates of overall kit mortality ranged from 4.5 to 10%, with one producer reporting simply that it was more than 50% higher than the previous year. Only one farm reported treating kits with antimicrobials.

The most common diseases reported (that is, specifically related to mortality, rather than a health problem not resulting in death) in post-weaned kits were heat stress, foot pad disease, hemorrhagic pneumonia, diarrhea, and dermatitis (‘sticky kits’). Only one producer reported increased mortality in adult females for 2013. The most common diseases reported in adult females (specifically related to mortality) were foot pad disease, hemorrhagic pneumonia (likely bacterial), and mastitis. Three farms provided an estimate of female mortality, which ranged from 0.5 to 1.5%. Two farms indicated that enrofloxacin was given to adult females; however, information on frequency of use was not provided.

#### Biosecurity responses

There were no significant differences in responses to the survey between those farms categorized as ‘small’ (<3400 females; 4/11) or ‘large’ (≥3400 females; 7/11). As the number of years a female was kept increased, the odds of kits having any abnormality identified at post mortem exam significantly decreased (OR = 0.42; 95% CI 0.19 to 0.92; P = 0.029). There was no effect of farm size or number of years in operation on biosecurity practices.

More than half of respondents did not feel that the mink industry had biosecurity standards in place and there were very few biosecurity and pre-emptive health management practices used uniformly by producers, with the exception of vaccinations. Three of 11 respondents indicated that no special biosecurity practices were in place on their farm. The use of personal protective equipment, policies on basic hand-washing and dedicated clothing were each implemented by fewer than 50% of respondents, while control of shed access, disinfection of feed containers after use and use of a rodent control program were the only practices implemented by greater than 70% of respondents (Tables 5, 6). Most producers (9 of 11; 81%; 95% CI 48.2 to 97.7%) used several methods for manure disposal. The most common methods for manure disposal (temporary vs permanent were not specified) were field spreading and an outdoor pile, with 8 of 11 (72%; 95% CI 39.0 to 94.0%) producers implementing each of these methods. Less commonly used methods included a temporary storage shed (1 of 11; 9%; 95% CI 0.2 to 41.3%), composting (3 of 11; 27%; 95% CI 6.9 to 61.0%), and hauling away from the farm (5 of 11; 45%; 95% CI 16.7 to 76.6%).

**Table 5:** Specific biosecurity protocols implemented by mink producers (n = 11)

Specific biosecurity practice	No. of producers utilizing specific practice
Foot baths	2
Restricted access to shed	3
Restricted access to property	6
Personal protective equipment (PPE)	4
Controlled traffic (i.e. young to old, clean to dirty)	1
Separate work washrooms	3
Designated clothes for sheds	3
Hand washing policies	1
Ante rooms	0
Locks on shed doors	0

**Table 6:** Summary of responses for biosecurity-specific questions on survey with yes/no answers (a) or multiple answers (b) (n = 11)

Survey question	Yes (%)	Exact 95% CI
(a)		
In your opinion, does CMBA^a^ or OFBA^b^ discuss biosecurity at meetings?	9/11 (81.8)	48.2 to 97.7
In your opinion, does industry have biosecurity standards?	5/11 (45.5)	16.7 to 76.6
Controlled access to sheds?	8/11 (72.7)	39.0 to 94.0
Controlled access to farm?	5/11 (45.5)	16.7 to 76.6
Employees wash hands		
Before entering sheds	0/11 (0)	0 to 28.5^c^
After leaving sheds	0/11 (0)	0 to 28.5^c^
Before handling mink	0/11 (0)	0 to 28.5^c^
After handling mink	5/11 (45.5)	2.3 to 51.8
Visitors wash hands		
Before entering sheds	0/11 (0)	0 to 28.5^c^
After leaving sheds	1/11 (9.1)	16.7 to 76.6
Before handling mink	0/11 (0)	0 to 28.5^c^
After handling mink	2/11 (18.1)	2.3 to 51.8
Restricted access to storage manure?	3/11 (27.3)	6.0 to 61.0
Employees trained to recognize disease or sickness?	7/11 (63.6)	30.8 to 89.1
Feed containers washed and disinfected after use?	8/11 (72.7)	39.0 to 94.0
Any integrated fly control programs used?	7/11 (63.6)	30.8 to 89.1
Companion animals have access to sheds?	9/11 (81.8)	48.2 to 97.7
Dedicated clothing for work in sheds?	5/11 (45.5)	16.7 to 76.6
Dedicated farm shoes?	5/11 (45.5)	16.7 to 76.6
Farm clothing washed separately?	7/11 (63.6)	30.8 to 89.1
Rodent control program on farm?	8/11 (72.7)	39.0 to 94.0
Birds have access to sheds?	11/11 (100)	71.5 to 1^c^
Wood used in pens?	11/11 (100)	71.5 to 1^c^
Mortality log kept?	4/11 (36.4)	10.9 to 69.2
Borrow males from other farms?	0/11 (0)	0 to 28.5^c^
Farm policies on traffic?	6/11 (54.5)	23.4 to 83.3
Mortality log kept?	4/11 (36.4)	10.9 to 69.2

Survey question	Response			
(b)				
No. of visitors to sheds per week	0 to 1: 10/11	2 to 4: 1/11		
Guests wear special clothing?	No: 7/11	Boots: 2/4	Boot covers: 1/4	Coveralls: 1/4
Storage of mink carcasses until disposal	None: 2/11	Freezer: 3/9	Solid container: 3/9	Compost: 3/9
Change storage between summer and winter?	Y: 3/11			
How often are dead animals collected?	Daily: 6/11	2 to 4×/week: 2/11	Biweekly: 1/11	N/A: 1
How are dead mink disposed of?	Composting: 9/11	Incineration: 1/11	Both: 1/11	
New mink brought on to farm?	Y: 7/11			
If so, quarantine?	No: 2/7	2 weeks: 1/7	1 month: 4/7	
New stock separate from main herd?	No: 2/7	Separate shed: 3/7	Same shed, different row: 2/7	
Type of ventilation	Natural: 8/11	Chimney: 3/11		
Kit pens cleaned	No: 3/11	W/water: 7/11	W/disinfectant: 1/11	
Female pens cleaned	No: 4/11	W/water: 5/11	W/disinfectant: 2/11	
Kit pens cleaned	No: 6/11	W/water: 4/11	W/disinfectant: 1/11	
Frequency waterlines are cleaned	Sporadically: 1/11	Yearly: 3/11	Rarely: 5/11	Weekly 2/11
Vaccinations given?	Distemper: 11/11	MEV: 10/11^d^	*Pseudomonas*: 10/11	Botulism: 10/11

Unless otherwise specified, questions pertain to the respondent’s specific on-farm practices

^a^Canada mink breeders association

^b^Ontario fur breeders association

^c^One-sided, 97.5% confidence interval

^d^Mink enteritis virus

## Discussion

Similar to previous studies [2, 3], the cause of death in the majority of mink kits examined grossly in this study was not determined. As kit weight increased, the odds of being born dead decreased and more than 50% of fetuses that were born dead were <11 g, suggesting that low kit birth weight may contribute to increased risk of early mortality. In 2013, the incidence of producer-reported mink kit preweaning mortality (of those born alive) ranged from 3 to 15%; environmental conditions were within those expected, and likely not contributing significantly to the incidence of preweaned kit mortality. Gross findings are not expected for a range of other infectious and noninfectious causes of preweaning mortality in kits, including various virus infections (kit or pregnant female), chilling, poor maternal care, lack of milk, and failure to thrive syndrome. A shortcoming of this study was the lack of statistical power. Had more producers participated in both parts of the study, additional associations between biosecurity practices and post mortem findings may have been identified.

Mink producers assisted with the development of national mink biosecurity guidelines which have been widely available to producers for close to a year at the time the survey was conducted [7, 15]. Despite this, only 45% of respondents in this study felt that the industry had adequate biosecurity standards. This was also reflected in the overall lack of consistency of implementation of recommended biosecurity practices by the producers surveyed in the second part of this study. To that end, the results of this study will provide a benchmark for the Canadian mink industry to assess areas in which greater attention should be given to enhance biosecurity practices. The industry may also use this as an opportunity to provide additional training and educational programs for producers.

Although certain enteric viral pathogens, such as mink enteritis virus (MEV) and epizootic catarrhal gastroenteritis of mink (a coronavirus) [17, 18], are known to cause significant morbidity and mortality in mink, the role of specific bacterial agents as a primary cause of preweaned kit enteropathy is not as well-established [19]. Samples from 45 of 69 kits with suspected enteritis were culture positive for at least 1 bacterial species. *Enterococcus* spp. were most common, with *E. faecalis* isolated from 43% of samples and *E. faecium* from nearly 20%. These agents are both common, commensal gastrointestinal tract bacteria of most mammals, including mink, and may not have been the primary cause of enteritis [20–22]. *Enterococcus gallinarum* was cultured in 3 of 69 samples and likely represents another commensal organism, as it has not previously been associated with disease in mink. Both *E. avium* and *E. hirae* are common gastrointestinal bacteria of birds, including poultry [23, 24]. Given the frequent use of poultry offal for mink feed this finding is not unexpected. Some strains of *E. coli* are thought to be a significant cause of morbidity and mortality in farmed mink and this bacterium has been implicated as a cause of hemorrhagic pneumonia, enteritis, mastitis, and septicemia [16, 17, 25]. However, *E. coli* has also been shown to have a high prevalence in apparently healthy adult female mink and their kits and further genotyping and toxin isolation is needed to determine the significance of isolated strains [22, 26, 27]. Additionally, when prevalence of *E. coli* serotype was investigated, there was no difference between healthy kits and those affected by sticky kit disease [27].

Of concern was identification of *Salmonella enterica* serovar Heidelberg from 11% (5 of 45) of culture positive samples, an agent of significant public health concern. This bacterium has been associated with food-borne disease outbreaks in Canada and the USA [28, 29]. Salmonellosis is usually food-borne, with poultry and pork products representing the most common sources of contamination [30]. *Salmonella* spp. infection has been linked to abortion and stillbirths in mink, dogs and cats [31–34]. Furthermore, although less common, an asymptomatic carrier state can also occur in humans and animals [35]. Less than 50% of producers (5/11) in this study reported that employees washed their hands after handling mink and only one producer had hand-washing policies in place. Thus, inadvertent zoonotic transmission from contaminated fecal matter could occur when poor hygiene practices are present, emphasizing again the importance of biosecurity practices on-farm.

*Staphylococcus delphini,* identified in 15% of gastrointestinal tract samples from kits with suspected enteritis and one skin sample, has previously been associated with an outbreak of ‘sticky kit’ syndrome with high mortality in the USA [36]. This syndrome presents as neonatal diarrhea characterized by mucoid feces and wet and sticky fur of affected kits [36, 37]. Both bacterial and viral agents have been isolated from affected kits in other studies, including astrovirus, coronavirus, mink enteric calicivirus, *S. delphini*, and *Salmonella* spp. [31, 36–39] and the precise etiology of ‘sticky kit’ syndrome is unknown. Although enteritis was not a common finding in the present study, outbreaks of neonatal diarrhea can be a significant cause of morbidity and mortality on mink farms and the etiology is likely multifactorial.

Lesions were present in 29% of kits examined in this study and the most common abnormality noted was excessive fluid accumulation, including hydrothorax, hydroperitoneum, subcutaneous fluid, and anasarca. Abnormal fluid distribution is a nonspecific clinical sign, which can arise from increased hydrostatic pressure, decreased oncotic pressure, increased vascular permeability or obstruction of fluid clearance. In dogs inoculated with minute virus of canines (a parvovirus), some dams had stillbirths or whelped pups with anasarca [40]. Further, causes of fading pups, pup abortion and pup stillbirth in dogs can include infection with canine herpes virus 1a [41–43] and canine parvovirus [40, 44]. Similarly, in cats, abortion, stillbirth and fading kittens have been linked to infection with feline panleukopenia virus (a parvovirus) or feline infectious peritonitis virus [45–51]. Infection with Aleutian mink disease virus, a parvovirus of significant concern in mink, has been shown to decrease conception rate, litter size and weight, and increase neonatal mortality, even in kits born to clinically healthy females [52]. Mink-specific viruses are less well characterized compared with those of domestic dogs and cats and their role in kit stillbirth, abortion, and abnormal development are largely unknown. Moreover, mink have been shown to be susceptible to infection with feline panleukopenia virus [53] and canine distemper virus [11, 54], which reinforces that companion animals should be excluded from mink sheds. Findings from this study suggest that more work is needed to characterize viral infections of mink and their impact on kit mortality.

A significant noninfectious factor in mink kit survival is the mothering ability of the female, particularly nest building, kit retrieval, and nursing [55]. Mink kits are dependent on the female for warmth and nutrition, as they are altricial at birth with an undeveloped thermoregulatory system and minimal fat stores [55–58]. Kits are especially susceptible to hypothermia and lose heat quickly during the first few days of life when exposed to cold [56, 57]. Females with higher kit survival rates spend significantly more time exhibiting kit-directed behavior [55]. Further, compared to kits from females provided with either straw as a nesting substrate, a plastic or artificial nest, or both, kits from females provided with only a nest box and wood shavings (the current standard on most Canadian mink farms) had significantly lower body weights 1 week after birth and higher mortality rates compared to the other groups [59]. Females provided with nesting material were also quicker to retrieve kits that had been removed from the nest [59]. This suggests that provision of supplemental nesting materials and selection of females for mothering abilities, a heritable trait in many mammals, could significantly decrease kit mortality and contribute to overall greater production yields and improved kit welfare.

In addition to human activity and companion animals as a source of pathogen introduction, wildlife pose a significant risk to mink health [8–12]. National mink biosecurity guidelines recommend the use of effective security fences, self-closing, lockable gates, and enclosed sheds to minimize wildlife access to mink [7, 15]. Just over half of respondents reported having fences around their mink sheds/farm, although information on the types of sheds used was not collected. Reasons for not having such measures were not collected, but cost is a possible explanation. The logic for implementing biosecurity standards, specifically in mink production, is evident in the near eradication of Aleutian disease virus in Denmark. Voluntary implementation of testing, quarantine, and limited movement of mink decreased the number of positive farms from 100% in 1976 to 15% in 1996; further reduced to <5% in 2001 following implementation of additional government-mandated biosecurity measures [9]. Even if the goal is not eradication of a specific disease from a geographical area, limiting the spread of potential pathogens and preventing outbreaks of infectious disease should represent a viable goal for the Canadian mink farming industry.

Despite mortality of preweaned kits being a significant cause of loss to producers, few studies have evaluated specific causes of death. In this study, we sought to estimate associations between management practices of farmers with causes of preweaned kit mortality, as well as characterizing the current state of biosecurity practices of the Canadian mink farming industry. Enhancing on-farm biosecurity practices as per national industry recommendations will assist with reducing infectious and contagious causes of mortality in mink kits, likely resulting in increased productivity and animal well-being.

## Additional file

**Additional file 1.** Biosecurity survey questions sent to mink producers.
