# Supplementary material for: On-farm biosecurity practices and causes of preweaning mortality in Canadian commercial mink kits
Source: Acta Vet Scand. 2017 Sep 8;59:57. doi: 10.1186/s13028-017-0326-8 (PMC5591539; doi:10.1186/s13028-017-0326-8)
Supplement: Supplementary file 1 — Additional file 1. Biosecurity survey questions sent to mink producers. [file 13028_2017_326_MOESM1_ESM.docx]

**Additional file 1:** Biosecurity survey questions sent to mink producers

Name (optional):_____________________________________________

County (optional):____________________________________________

**General Farm Information**

**1)** What is the size of your farm (total # of female mink): _________________

**2)** How long has your mink farm been in operation? _____________________

**3)** How many employees work on your farm? (Please include any family members who participate in chores) **a)** full time: ___________ **b)** part time (summer): _____________ c) part time (pelting)

**4)** Total number of pelts produced in 2013: ______________________________

**5)** Total number of kits born in 2013: ___________________

**6)** On average, how many years are breeding females kept? ___________

**7)** On average, how many years are breeding males kept? _____________________

**8)** Do you own other livestock? YES NO

1. If so, what kind(s)? (list all)____________________________________________

**b)** On the same property as the mink? YES NO

**c)** Same sheds? YES NO

**9)** What types of livestock farms are within a 3km radius of your main mink operation?

(Check all that apply)

Swine

Chickens

Dairy

Beef

Turkey

Mink

Other (please specify)­­­­: _________________________

None

**10)** How often do you visit other livestock farms?

Daily

1-3 times weekly

Monthly

Less than monthly

Never

11) Visitors (including neighbours) to your farm are from which types of farms?

(Check all that apply)

Swine

Chickens

Dairy

Beef

Turkey

Mink

Other (please specify)­­­­: _________________________

None

**12)** What is the source of water for your mink farm? (Check all that apply)

Well water

Deep (drilled) well

Shallow (dug) well

Cistern

Rain water

Municipal water

Surface water (river, stream, pon)

Other (please specify): _________________________

**13)** Is the water tested for bacteria? YES NO

If yes, how often?

Quarterly Annually Less frequently than annually

**14)** Do you treat the water (ie, filter, chlorination)? YES NO

If yes, how? (List all methods of treatment that apply):

**15)**  If you use a filter, how often is the filter changed

Daily

Weekly

Bi-weekly

Monthly

Yearly

Never

**16** ) Is there a fence surrounding your mink sheds/farm? YES NO

**17) (Please skip this question if you answered NO to question 16.)**

If a fence is present is it:

at least 6 feet high? YES NO

**18)** What type of diet are your mink fed (check all that apply)?

Pellets Wet feed (ready made)

Wet feed (made on-farm)

**19**) Are the floors of the mink sheds

Packed soil Cement Other (Please specify)___________________

**Animal Health**

**20)** Was there increased mortality (death) in your **kits** prior to weaning in 2013 compared to previous years? YES NO , after weaning YES NO , both times YES No

**a)** Kit signs (eg. diarrhea, sneezing, etc):____________________________________________

**b)** Estimated mortality (%):______________________________________________________

**c)** What was/were the treatment(s):______________________________________________

**d)** How effective was/were the treatment(s) in reducing mortality:

VERY GOOD , GOOD FAIR POOR

**21)** Was there an increased mortality in your **females** in 2013 compared to previous years? YES NO

**a)** Female signs (eg. diarrhea, sneezing, etc):________________________________________

**b)** Estimated mortality (%):______________________________________________________

**c)** What was/were the treatment(s): ______________________________________________

**d)** How effective was/were the treatment(s) in reducing mortality: _____________________

**22)** What are the most common health problems seen in the **kits** on your farm? (Check all that apply)

Diarrhea

Respiratory

Neurologic

Weight loss

Feed refusal

Unexplained death

Ear/fur mites

Other: _________________

**23)** What are the most common health problems seen in the **females** on your farm? (Check all that apply)

Diarrhea

Respiratory

Neurologic

Weight loss

Feed refusal

Unexplained death

Ear/fur mites

Other: __________________

**24)** Do you use antibiotic treatment for diseases of bacterial origin?

YES NO If so, what common antibiotic(s) do you use (Please list all)?

**25)** Do you incorporate antibiotic(s) in the feed/water supply on a regular basis?

YES NO If so, what antibiotic(s) do you use (Please list all)?

**26)** How often are antibiotics administered to mink?

Daily

Semi-weekly (1-3 times per week)

Bi-weekly (every other week)

Not applicable (i.e. – do not use antibiotics)

Other (such as seasonally) (Please specify when or for what indication):

**Biosecurity**

**27)** Does the CMBA or OFBA discuss issues related to biosecurity at its meetings? YES NO

**28)** In your opinion, does the industry have biosecurity standards? YES NO

**29)** Do you have farm policies on human and vehicular traffic (ie, designated parking):YESNO

**30)** Is access to the sheds controlled? YES NO

**31)** Is access to the farm controlled? YES NO

**32)** How many visitors do you get in your sheds per week?

0-1

2-4 >5

**33)** Do guests have to wear special clothing or follow special procedures when entering your farm/sheds?

YES NO **(if Yes, Please check any that apply)**

Boots

Boot covers

Coveralls

Hand washing

Visitors sign in log book

Other, please explain:

**34)** Do you have dedicated clothes for working in the sheds? YES NO

**35)** Do you have dedicated work boots/shoes worn in the sheds? YES NO

**36)** Do you follow any biosecurity protocols (check all that apply)?

Foot bath

Ante room (a smaller room serving as an entryway into a larger room)

Locks on shed doors

Restricted access to sheds

Gate or restricted property access (i.e. - perimeter fence)

Personal protective equipment (boots, etc)

Traffic movement from young to old mink or from clean to dirty areas

Separate work washroom,

Designated clothes for in sheds

Designated shed equipment

Cleaning and disinfection standards

Hand-washing policies

**37)** Is farm clothing washed separately from non-farm laundry? YES NO

**38)** Do employees wash their hands before:

**a)** Entering the shed(s)? YES NO

**b)** Leaving the shed(s)s? YES NO

**39)** Do visitors wash their hands before:

**a)** Entering the shed(s)? YES NO

**b)** Leaving the shed(s)? YES NO

**40)** Do employees wash their hands:

**a)** Before handling mink? YES NO

**b)** After handling mink? YES NO

**41)** Do visitors wash their hands:

**a)** Before handling mink? YES NO

**b)** After handling mink? YES NO

**c)** N/A, visitors do not handle the mink YES NO

**42)** How do you store mink carcasses (deadstock) until they are collected or disposed of?

Freezer

Solid container

N/A, carcasses are not stored

Other (Please specify) : _______________________________________

**43)** Does the storage of carcasses (deadstock)change between summer and winter months?

YES NO If yes, how (please explain): _________________________________

**44)** On average, how often are dead animals collected from this farm?

Daily

2-4 times weekly

0-1 times weekly

Bi-weekly

N/A

Other (Please specify)

**45)** How do you dispose of dead mink on your farm?

Composting

Incineration

Burying

Rendering/deadstock collector

City disposal/regular garbage

Other (Please specify):

**46)** Do you keep a mortality log? YES NO

**47)** Are all employees trained to recognize disease or sickness in the mink?

YES NO

**48)** Do you bring new mink into the farm? YES NO

**49)** Do you have quarantine practices or procedures for new mink? YES NO

If yes, how long is new stock quarantined for: _______________

**50)** Is new stock kept separate from main herd? YES NO

**a)** in a separate shed? YES NO

**b)** in the same shed but in a different area (i.e. row)? YES NO

**51)** Do you borrow/share males with another farm? YES NO

If yes, what, if any, special quarantines practices do you follow (if any)?

**52)** What type of ventilation system do you have for your mink operation?

(Please check all that apply)

Negative pressure (mechanical with fans)

Chimney ventilation

Natural ventilation

Evaporative cooling (i.e. – misting)

Recirculation ventilation

Other (Please specify):

**53)** What is your method of manure/litter disposal? (Check all that apply)

Temporary shed storage

Compost

Outdoor pile

Hauled off farm

Spread on fields on farm

Other (Please specify)

**54)** Is there restricted access to the stored manure? YES NO

**55)** Where do you store the mink feed/feed ingredients?

Dry ingredients: _____________________________

Meat/dairy/fish byproducts: ____________________

Finished feed: ________________________________

Minimal on-farm storage: _______________________

**56)** Is there a rodent control program on your farm? YES NO If yes, please specify:

**57)** Is wood used in any portion of your mink pens or nestboxes? YES NO

**60)** Are

____

**58)** Are the **kit** pens cleaned and disinfected? YES NO

If yes, check any that apply:

Cleaned of debris with water

Cleaned of debris with water and soap

Cleaned then disinfected with a chemical/disinfectant

Other (Please Specify): _______________________

**59)** Are the fe**male** pens cleaned and disinfected? YES NO

If yes, check any that apply:

Cleaned of debris with water

Cleaned of debris with water and soap

Cleaned then disinfected with a chemical/disinfectant

Other (Please Specify): _______________________

**60)** Are the **males** pens cleaned and disinfected? YES NO

If yes, check any that apply:

Cleaned of debris with water

Cleaned of debris with water and soap

Cleaned then disinfected with a chemical/disinfectant

Other (Please Specify): _______________________

**61)** How often are waterlines/nipples flushed, cleaned and/or disinfected?

Weekly

Monthly

Yearly

Rarely

Sporadically/irregularly

**62)** Are any integrated **fly** control programs in effect?

YES NO

If **YES**, what methods are used (Please check all methods that apply)?

cement floors drainage systems around farm

regular manure manure piles are tarped

parasitic nematodes are added to manure piles

liquid manure system


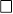
 manure is composted

other (please specify)______________________________________________

**63)** Do companion animals (i.e. dogs, cats) have access into the mink sheds? YES NO

**64)** Do birds have access into the mink sheds? YES NO

**65)** Spread of disease is minimized by working with animals from **youngest to oldest** and **healthy to sick**. Does your farm implement these practices when handling your mink?

YES NO

**66)** Are feed containers/totes washed and disinfected thoroughly after each use?

YES NO

**67)** Are vaccinations performed? YES NO

Please check off the diseases mink are vaccinated for and indicate age when vaccinated

Distemper at age of ___ weeks virus enteritis at age of ___ weeks

pseudomonas pneumonia at age of ___ weeks botulism at age of ___ weeks

other (please specify) ___________________________________

Are there any other notes or comments about production on your operation that you would like to make:________________________________________________________________________________________________________________________________________________________________________________________________________________________________________

Thank-you for completing this survey!
